# Dynamic temporal partitioning enhanced transformer for pediatric viral load forecasting

**DOI:** 10.3389/fpubh.2026.1829861

**Published:** 2026-06-05

**Authors:** Sai Li, Zhengqiu Li, Yi Mo, Sitian Chen, Zeshu Ning, Junkai Ren, Xiaozhou He

**Affiliations:** 1 Department of Clinical Laboratory, The Affiliated Children's Hospital of Xiangya School of Medicine, Central South University, Hunan Children's Hospital, Changsha, China; 2 Department of Neurology, The Affiliated Children's Hospital of Xiangya School of Medicine, Central South University, Hunan Children's Hospital, Changsha, China; 3 National Key Laboratory of Intelligent Tracking and Forecasting for Infectious Diseases, National Institute for Viral Disease Control and Prevention, Chinese Center for Disease Control and Prevention, Beijing, China

**Keywords:** dynamic temporal partitioning, pediatric infectious disease monitoring, pediatric viral load prediction, sparse self-attention, time series analysis, transformer

## Abstract

**Introduction:**

Viral infectious diseases are highly prevalent in pediatric populations, and accurate prediction of viral load is critical for clinical intervention and public health management. Existing time-series models poorly handle long-range dependencies, multi-scale features, noise, and incomplete clinical data.

**Methods:**

We propose DTR-Former, a Dynamic Temporal Partitioning-enhanced Transformer. It uses wavelet packet decomposition for adaptive temporal partitioning and multi-scale feature extraction, adopts sparse self-attention to reduce redundancy and enhance long-sequence modeling, and employs a residual convolutional decoder with a gating mechanism to refine features and suppress residual noise.

**Results:**

On dbEBV dataset, DTR-Former achieves MSE = 0.16, MAE = 0.27, *R*^2^ = 0.88; on NCBI dataset, MSE = 0.18, MAE = 0.30, *R*^2^ = 0.86. It shows strong multi-step stability and robustness under missing-data conditions.

**Conclusion:**

DTR-Former outperforms state-of-the-art methods in accuracy, stability, and efficiency, offering an effective solution for pediatric viral load forecasting and related time-series tasks.

## 1 Introduction

The dynamic variation in viral load can accurately reflect the replication patterns and temporal evolution of viruses in the body. Accurate prediction of pediatric viral load is an important research direction in pediatric medical time series analysis, with significant academic value for research on the pathological mechanisms of virus-related diseases in children and for optimizing time series modeling methods for pediatric clinical data. In research on pediatric viral load prediction, the core task is to explore the characteristics of nonlinearity, multiscale fluctuations, and temporal dependence using historical time-series detection data from children, thereby enabling effective inference of future viral load values for pediatric subjects (1, 2, 48).

Research on viral load time series prediction has evolved from traditional time series modeling to deep learning. A large number of studies have been conducted to capture time-series features and improve prediction accuracy using various research methods at different stages. Early studies mainly relied on traditional time series analysis methods, with the ARIMA model and its improved variants as the core (3, 4). These methods capture the temporal trend in viral load under linear assumptions, offering a simple structure and high computational efficiency. They can achieve basic time-series prediction in scenarios with relatively stable data distributions, laying a methodological foundation for subsequent pediatric viral load prediction research (5).

With the rise of deep learning, research on viral load prediction has gradually shifted toward nonlinear time-series modeling, and recurrent neural networks such as LSTM and GRU have become the mainstream methods (6, 7). Such models initially capture temporal dependence through gating mechanisms, which, to a certain extent, address the nonlinear adaptation problem of traditional methods. They can better adapt to dynamic fluctuations in viral load (8, 9). They have achieved better performance than traditional methods on viral load prediction tasks across various virus types. Since then, researchers have further optimized the structure of recurrent neural networks and improved the model's ability to capture key time-series nodes by introducing attention mechanisms and feature fusion strategies, thereby gradually improving viral load prediction accuracy (10, 11). However, these models lack targeted optimization for the unique characteristics of viral load fluctuations in children with immature immune systems.

In recent years, leveraging the advantages of the self-attention mechanism, the Transformer model has been widely applied to time-series prediction, gradually replacing recurrent neural networks and emerging as a research hotspot in viral load prediction (12). Most of the existing relevant studies based on Transformers adopt the standard full self-attention mechanism, which can effectively model long-sequence dependencies and overcome the limitation of insufficient long-sequence dependence modeling in recurrent neural networks, showing good application potential in long time series viral load prediction tasks (13). Researchers have carried out preliminary adaptation of the Transformer model according to the characteristics of viral load data, and further improved the prediction performance of the model through data preprocessing optimization and network parameter tuning (14), but few studies focus on the adaptation of the model for pediatric viral load data with high volatility and short monitoring sequence characteristics. The relevant research findings offer new insights into the application of time series prediction methods in pediatric medicine.

Existing viral load time-series prediction methods still have limitations, and their applicability to pediatric viral load prediction is further restricted. Traditional methods cannot adapt to nonlinear time-series features, and recurrent neural networks have insufficient ability to model long sequence dependence (15, 16). Existing Transformer-based models suffer from computational redundancy, inaccurate feature extraction, and insufficient feature enhancement, making it difficult to meet the research requirements for accurate pediatric viral load prediction.

Aiming at the above research limitations, the research objective of this study is to propose a time series prediction model that adapts to the temporal characteristics of pediatric viral load and balances prediction accuracy and computational efficiency, to solve the shortcomings of existing methods in long sequence modeling, multi-scale feature capture, and feature utilization rate for pediatric viral load data. To this end, this study proposes a temporal Transformer model based on dynamic temporal partitioning and residual feature enhancement for pediatric viral load forecasting. The core design includes three modules: the dynamic temporal partitioning module realizes the structured partitioning of pediatric viral load time series based on wavelet packet decomposition to accurately extract multi-scale temporal features; the sparse self-attention Transformer module reduces computational redundancy and strengthens the modeling ability of long sequence temporal dependence by limiting the scope of attention calculation; the residual feature enhancement decoder integrates convolutional and residual networks to realize the enhancement of feature expression and accurate regression prediction for pediatric viral load.

The main contributions of this study are as follows:

- A dynamic temporal partitioning module is designed, which completes the multi-scale structured partitioning of pediatric viral load time series through wavelet packet decomposition, effectively separates noise from valid features, and improves the utilization rate of temporal features for pediatric clinical monitoring data.
- The sparse self-attention mechanism is introduced to optimize the Transformer architecture, which solves the computational redundancy problem of the standard Transformer and improves the modeling efficiency and accuracy of long sequence temporal dependence for pediatric viral load time series.
- A residual feature enhancement decoder is constructed. Through the integration of convolutional operations and residual connections, the decoder strengthens feature representation, reduces feature degradation, and thereby improves the accuracy and robustness of pediatric viral load regression prediction.

The rest of this study is organized as follows: Chapter 2 reviews related studies and summarizes research progress in pediatric viral load prediction and in temporal Transformer-based models with feature enhancement. Chapter 3 elaborates on the overall architecture of the model and the design details of each core module for pediatric viral load forecasting. Chapter 4 verifies the effectiveness of the model through experiments on pediatric viral load datasets and compares its performance against baseline models. Chapter 5 discusses the experimental results, model limitations, and future research directions in depth for pediatric viral load prediction scenarios. Chapter 6 summarizes the research content and core conclusions of the full text.

## 2 Related work

### 2.1 Research progress on viral load time series prediction

With the accumulation of large-scale epidemic data, machine learning and deep learning methods have attracted extensive attention for predicting viral load and related time series data. A growing number of studies hold that simple linear models are difficult to capture the complex characteristics of viral load data, such as nonlinearity, multi-scale variation, and noise interference; thus, exploring more advanced deep architectures has become a research hotspot (17). For example, in predicting Coronavirus disease of 2019 (COVID-19) transmission trends and cases, studies have compared various deep learning structures, including hybrid models integrating CNN/LSTM and Transformer variants models (18). The results show that deep models can effectively capture nonlinear dynamic characteristics and achieve significantly better performance than traditional autoregressive models. Some studies have further introduced ensemble learning strategies to fuse the prediction results of different models for improving stability and accuracy, and such hybrid modeling methods have shown stronger generalization ability in the trend prediction of different diseases (19–21).

In the field of pediatric medical time series analysis, scholars have combined LSTM with attention mechanisms to enhance the ability to capture long-term dependencies and proposed a gated attention fusion structure for pediatric viral load fluctuation data with strong noise interference. Furthermore, some studies have used a combined CNN-RNN architecture to extract local time-series patterns and fuse them with global dependencies (22, 23). These methods have achieved better results than pure LSTM on pediatric disease course datasets with severe fluctuations. Simultaneously, to solve the gradient attenuation problem of recurrent networks in long sequence modeling for pediatric viral load data, some studies have introduced residual connections and hierarchical structures to enhance the expressive ability of traditional time series networks (24, 50). More recent studies have begun to introduce the Transformer architecture into pediatric medical time series prediction tasks (25–27). Since pediatric viral load sequences usually involve long time span change trends and short-term fluctuations characteristic of children with immature immune systems, the self-attention mechanism of the standard Transformer can capture global dependencies, thus achieving good prediction results. However, these studies also point out that when dealing with pediatric medical time series data with multi-scale and strong noise, the standard Transformer has the problems of high computational complexity, attention redundancy, and insufficient local feature capture ability. To address these problems, many studies have improved the Transformer architecture by fusing convolutional modules and multi-scale feature extraction modules to enhance its modeling ability for complex pediatric biomedical sequences (28, 29).

Although the above studies have made remarkable progress in time series prediction, existing methods still struggle to simultaneously balance the modeling of long sequence global dependencies, the capture of multi-scale dynamic characteristics, and the ability of noise interference suppression for pediatric viral load data. The dynamic temporal partitioning module proposed in this study combines wavelet packet decomposition to realize multi-scale decomposition of pediatric viral load time series data, and reduces computational redundancy through the sparse self-attention mechanism, thus extracting noise and valid features in pediatric viral load sequences more precisely and improving the accuracy and stability of prediction.

### 2.2 Temporal transformer and feature enhancement

Since its introduction, the Transformer model has attracted extensive attention in the field of time series prediction. Its architecture, based on the self-attention mechanism, can identify dependencies between any positions within the sequence, a feature that is particularly important in long sequence prediction tasks. Recent large-scale reviews have pointed out that in long-term time series forecasting tasks, various Transformer variants have become among the most successful model frameworks, and a variety of optimization strategies have emerged to address the challenges of long sequence modeling (30).

One category of research focuses on improving the computational efficiency of the self-attention mechanism. For example, Surrogate Attention Blocks and Surrogate FFN Blocks, which introduce structured attention to replace the traditional self-attention mechanism, not only significantly reduce the spatial and temporal complexity, but also improve the prediction performance on multiple long sequence tasks (31–33). Such structural optimizations are of great significance for large-scale pediatric viral load time series data. Another category of research, such as Skip-Timeformer, improves the local semantic modeling ability of Transformer in periodic sequences by performing multi-interval decomposition of time series and constructing token encoding methods with different time intervals (34, 35, 49). Furthermore, some studies have attempted to integrate graph structures or memory mechanisms into the Transformer to enhance dynamic correlation modeling of pediatric viral load data. For example, the Memformer architecture captures local and global information by combining patch-wise graph learning and global attention, improving the model's ability to identify dynamic correlations and its robustness (36, 37). Other studies have proposed modular structures such as sTransformer that combine sequence convolution with attention mechanisms to simultaneously improve the ability to capture cross-sequence and intra-temporal features of pediatric viral load data (38–40). These works demonstrate the important role of feature enhancement and structural design in improving the temporal prediction ability of the Transformer for pediatric viral load data. Although these improvements have achieved good results in long-sequence prediction, most current Transformer variants still have two types of deficiencies when applied to pediatric viral load prediction. First, the fusion of multi-scale information is insufficient, especially when it is necessary to handle global trends and high-frequency fluctuations common in pediatric viral load data simultaneously; it is difficult to consistently obtain high-quality features (41, 42). Second, noise interference and redundant information weaken the effective learning ability of the model, especially when pediatric viral load data contain many meaningless or weakly periodic components; the impact is significant.

To address the above problems, the sparse self-attention mechanism proposed in this study limits the scope of attention calculation to reduce redundancy and combines convolutional residual networks as the residual feature enhancement decoder. Enhancing the local expression ability for pediatric viral load data alleviates feature degradation, improves the model's expression of multi-scale features and noise robustness, and thus enhances the overall performance of long-sequence prediction for pediatric viral load.

## 3 Methodology

### 3.1 Overall architecture of the DTR-former model

**Figure 1:**
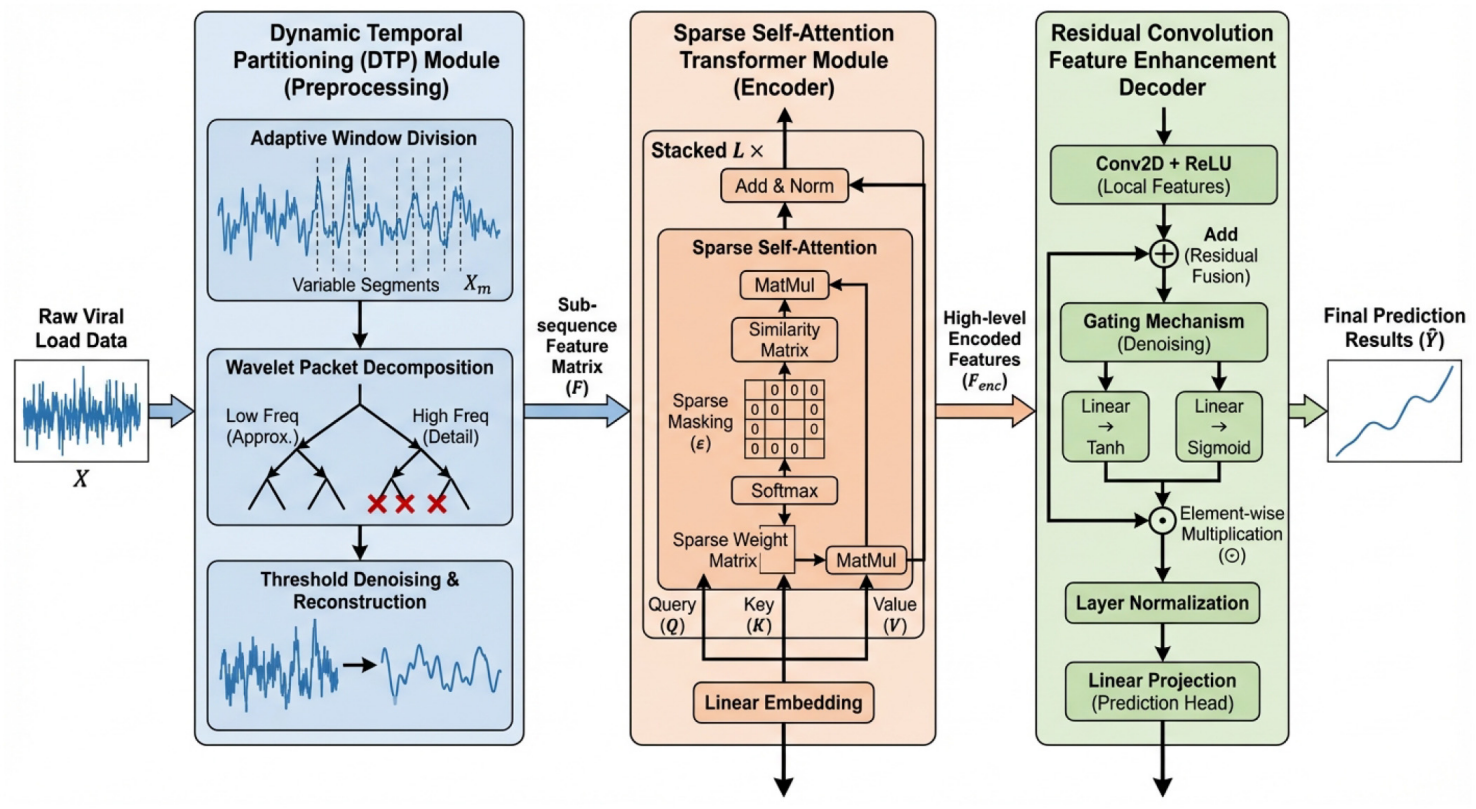
Overall architecture of the DTR-Former model comprising three core modules: Dynamic Temporal Partitioning preprocessing, Sparse Self Attention Transformer encoder, and Residual Convolution Feature Enhancement decoder for pediatric viral load prediction.

To address the challenge that existing viral load time series prediction methods struggle to simultaneously balance long sequence global dependency modeling, multi-scale dynamic feature capture, and noise interference suppression, especially for pediatric viral load data with high volatility, short monitoring sequences, and inherent noise from clinical sampling, this study proposes a time series prediction model integrating dynamic temporal partitioning and sparse self-attention, namely DTR-Former. Through a modular design, the model achieves accurate extraction, efficient modeling, and end-to-end prediction of complex features in pediatric viral load sequences, and its overall architecture is illustrated in Figure 1. The model employs a series of modular components that work collaboratively to perform end-to-end mapping from raw pediatric viral load data to final predictions, without requiring additional prediction-related modules. Specifically, raw pediatric viral load data is first fed into the preprocessing module. This module adaptively divides time windows based on the fluctuation characteristics of the pediatric viral load sequence and decomposes the original sequence into multi-scale subsequences using wavelet packet decomposition. This process initially separates effective features from noise, generating a subsequence feature matrix as the output. Subsequently, this feature matrix is input into the encoding module. By introducing sparse constraints to limit the range of attention computation, the module retains the ability to capture global dependencies while significantly reducing computational complexity, which is well-suited to the small-sample characteristics of pediatric clinical data. It further extracts long-term trends and short-term fluctuations of the sequence through hierarchical attention stacking, outputting high-level feature vectors. The encoded high-level features are then transmitted to the Residual Convolution Feature Enhancement Decoder module, which simultaneously performs feature enhancement and final prediction. This module integrates the advantages of local feature extraction from convolutional neural networks and the gradient-protecting characteristics of residual connections, effectively capturing local correlation information, alleviating gradient attenuation, and suppressing residual noise via a gating mechanism, thereby adapting to the high-noise nature of pediatric viral load sampling data. After feature refinement and enhancement, the module directly performs feature mapping using a combination of fully connected layers and activation functions, and outputs final pediatric viral load predictions according to specific pediatric clinical task requirements, such as short-term single-point prediction or long-term multi-step prediction. Overall, through the collaboration and complementarity of its modular structures, the DTR-Former effectively overcomes the shortcomings of existing methods, balances comprehensive feature capture with prediction accuracy and training efficiency, and thus provides a reliable and efficient modeling solution for pediatric viral load time series prediction.

### 3.2 Dynamic temporal partitioning module

**Figure 2:**
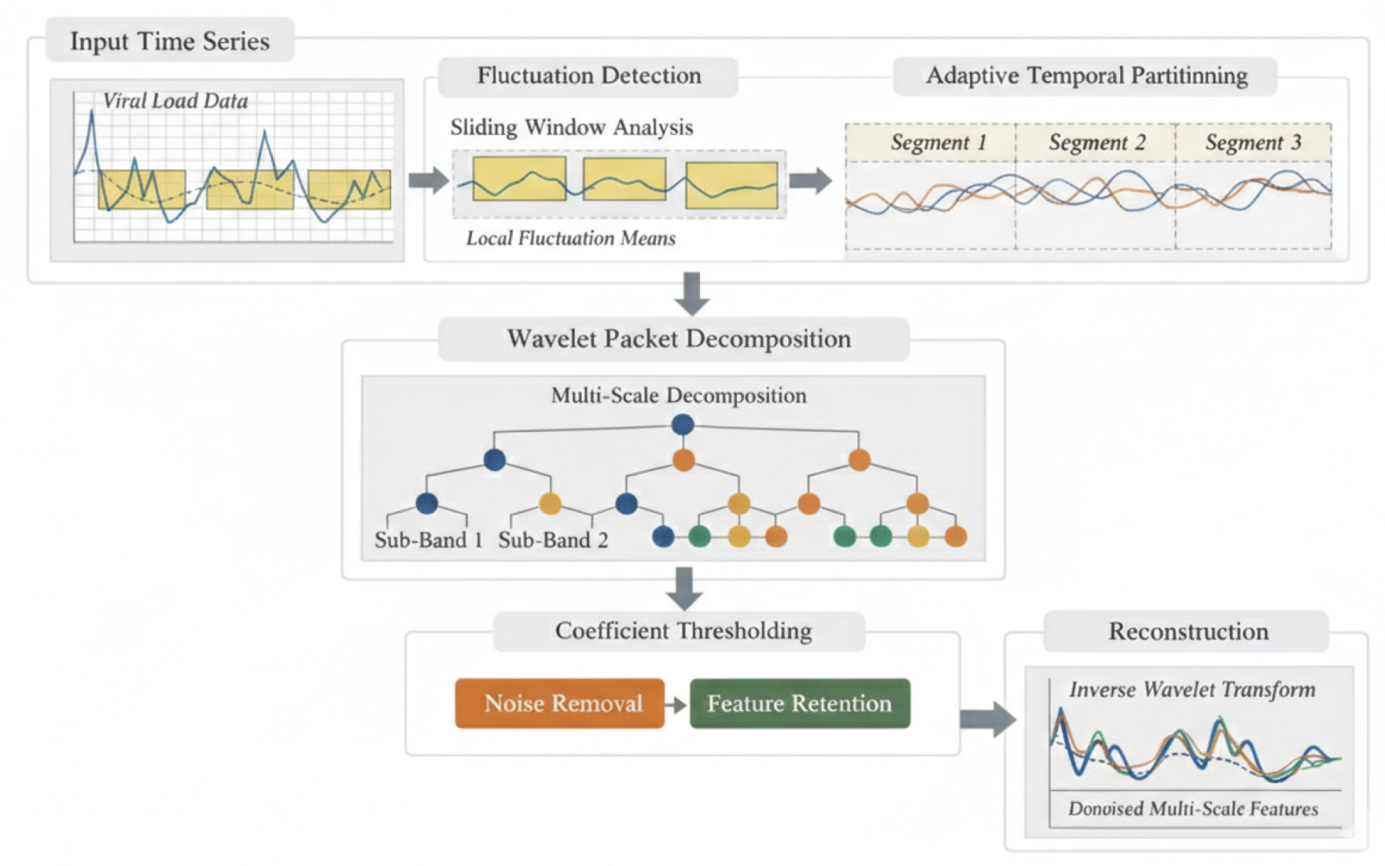
Workflow of the Dynamic Temporal Partitioning module for pediatric viral load data, which includes fluctuation detection.

The Dynamic Temporal Partitioning module is the core of feature preprocessing in the DTR-Former model. Its main function is to adaptively divide time windows and complete multi-scale decomposition according to the fluctuation characteristics of pediatric viral load time series data characterized by high volatility and short monitoring sequences in children, initially separating effective features from noise and providing high-quality input for the subsequent encoding module. Abandoning the limitations of traditional fixed-length partitioning, this module adaptively determines the size of partitioning windows by calculating the sequence fluctuation intensity and then performs multi-scale decomposition on the sequences within each window, combined with wavelet packet decomposition. For pediatric viral load data the sliding window length *w* is adjusted from the general setting to 5–7 adapted to short time span pediatric monitoring data and the fluctuation threshold λ is increased by 15% to enhance the capture of sudden fluctuation mutations in pediatric viral load. The setting of sliding window length from 5 to 7 is theoretically designed according to the inherent short sequence property of clinical pediatric viral load monitoring data, which guarantees sufficient local fluctuation statistical samples while avoiding oversize windows that smooth out real transient variation characteristics. The 15% upward adjustment of fluctuation threshold λ is theoretically designed for the high volatility and frequent abnormal jitter interference of pediatric viral load sequences, which aims to filter tiny redundant fluctuation noise and retain effective mutation boundaries of real viral load variation. The entire process is gradually realized through a series of coherent mathematical formulas. The overall workflow of the Dynamic Temporal Partitioning module is illustrated in Figure 2. The relevant formulas for this section are given in Equations 1–5.

First, the original pediatric viral load time series is defined as *X* = [*x*_1_, *x*_2_, …, *x*_*n*_] where *n* is the length of the sequence and *x*_*i*_ represents the observed pediatric viral load value at the *i*-th moment, e.g., viral load from nasopharyngeal swabs of children. To quantify the fluctuation intensity of the sequence at different moments the absolute value of the load difference between adjacent moments is calculated and the local fluctuation mean is computed based on a sliding window optimized for pediatric data with *w* = 5 − 7 which is used as the basis for partitioning. The calculation formula is as follows:


$$\begin{array}{l} \mu_{k}=\frac{1}{w}\times \overset{k}{\underset{i=k-w+1}{\sum }}|x_{i}-x_{i-1}| \end{array}$$ (1)

where μ_*k*_ denotes the local fluctuation mean at the *k*-th moment, *w* is the length of the sliding window adaptively adjusted to 5–7 for pediatric viral load datasets to fit short monitoring sequences, and |*x*_*i*_−*x*_*i*−1_| represents the fluctuation amplitude of pediatric viral load between adjacent moments. This formula smooths local fluctuations using a sliding window, avoiding the interference of abnormal values at a single moment, which is common in pediatric clinical sampling, on the partitioning results.

Based on the local fluctuation mean μ_*k*_, a fluctuation threshold λ is set to 15% higher for pediatric data to capture high volatility. Unlike conventional segmentation strategies that seek to minimize intra-segment fluctuations, our partitioning logic focuses on identifying clear mutation boundaries in viral load variation. Excessively pursuing minimal internal fluctuation would lead to over-segmentation of stable sequential segments and destroy continuous temporal correlation inherent in pediatric viral load sequences. Selecting the position with fluctuation closest to the threshold can divide the original sequence into sub-segments with relatively stable internal variation trends, which is more suitable for subsequent multi-scale wavelet packet feature decomposition and denoising processing. When the local fluctuation mean at a certain moment exceeds the threshold, that moment is determined as a sequence fluctuation mutation point, which is used to divide the time windows. The formula for determining the window boundary is:


$$\begin{array}{l} b_{m}=\arg\underset{k}{\min}\left\{\left|\mu_{k}-\text{λ}\right|\right\}\;\left(k\in \left[b_{m-1}+1,n\right]\right) \end{array}$$ (2)

where *b*_*m*_ denotes the right boundary of the *m*-th time window and *b*_0_ = 0. This formula finds the moment corresponding to the fluctuation mean closest to the threshold, thereby realizing adaptive partitioning of time windows for pediatric viral load sequences with high volatility. Finally, the original sequence *X* is divided into *M* non-overlapping sub-sequences *X*_*m*_ = [*x*_*b*_*m*−1_+1_, …, *x*_*b*_*m*__] (*m* = 1, 2, …, *M*) and each sub-sequence corresponds to a segment of pediatric viral load data with similar fluctuation characteristics.

To further separate effective features from noise caused by immature immune systems and sampling errors, in children, wavelet packet decomposition is performed on each adaptively partitioned sub-sequence *X*_*m*_. The subsequence is decomposed into components of different frequencies through multi-scale decomposition, and an appropriate wavelet basis function ψ(*t*) is selected db4 wavelet basis is preferred for pediatric viral load data to balance denoising and feature retention. The recursive formula of wavelet packet decomposition is:


$$\begin{array}{l} d_{m,j}\left(\tau \right)=\underset{k}{\sum }h\left(k\right)d_{m-1,j}\left(2\tau -k\right) \end{array}$$ (3)

$$\begin{array}{l} c_{m,j}\left(\tau \right)=\underset{k}{\sum }g\left(k\right)d_{m-1,j}\left(2\tau -k\right) \end{array}$$ (4)

where *d*_*m, j*_(τ) and *c*_*m, j*_(τ) represent the wavelet packet detail coefficient and approximation coefficient of the *j*-th node in the *m*-th layer respectively *h*(*k*) and *g*(*k*) are the low-pass filter and high-pass filter coefficients of wavelet packet decomposition respectively and τ is a continuous time variable. Through recursive decomposition, this formula separates the high-frequency noise components corresponding to the detail coefficient *d*_*m, j*_ mainly from pediatric sampling errors and low-frequency effective features corresponding to the approximation coefficient *c*_*m, j*_ reflecting real viral load changes in children of the sub-sequence.

Finally, threshold processing is applied to the decomposed wavelet packet coefficients to suppress high-frequency coefficients corresponding to noise, using a stricter threshold for pediatric data to reduce sampling noise and retain the low-frequency coefficients corresponding to effective features. The sub-sequence features are reconstructed via an inverse wavelet packet transform to obtain the denoised multi-scale sub-sequence feature matrix for pediatric viral load data. The inverse transformation formula is as follows:


$$\begin{array}{l} d_{m-1,j}\left(\tau \right)=\underset{k}{\sum }h\left(k\right)d_{m,j}\left(\tau /2-k\right)+\underset{k}{\sum }g\left(k\right)c_{m,j}\left(\tau /2-k\right) \end{array}$$ (5)

Through this inverse transformation formula, the processed coefficients are reconstructed into feature sequences with the same length as the original sub-sequences. Finally, the feature matrix reconstructed from all sub-sequences is output as the input of the sparse self-attention encoding module, completing the entire process of dynamic temporal partitioning and multi-scale denoising for pediatric viral load time series.

### 3.3 Sparse self attention transformer module

The Sparse Self Attention Transformer module is the core encoding unit of the DTR-Former model. Its main function is to perform global dependency modeling on the multi-scale subsequence feature matrix output by the DTP module. While effectively capturing long-term trend and short-term fluctuation patterns in pediatric viral load sequences, it addresses the high computational complexity and attention redundancy of the standard self-attention mechanism. It is optimized for the small-sample, high-volatility characteristics of pediatric clinical data. By introducing a sparse constraint strategy to optimize the attention computation, the model's encoding efficiency and feature-extraction accuracy for pediatric viral load features are improved. Building on the standard Transformer encoding structure, this module retains the advantage of hierarchical attention stacking while optimizing the self-attention computation path; its core logic is gradually realized through a series of progressively defined mathematical formulas. The relevant formulas for this section are given in Equations 6–11.

First, the multi-scale subsequence feature matrix of pediatric viral load output by the DTP module is defined as *F* ∈ ℝ^*M*×*d*^ where *M* is the number of adaptively partitioned sub-sequences and *d* is the feature dimension of each sub-sequence. This feature matrix is used as the input of the module, and the query matrix *Q*, key matrix *K*, and value matrix *V* are generated through linear transformations, respectively, to realize the unified mapping of feature dimensions and meet the input requirements of self-attention calculation for pediatric viral load data. The calculation formulas are:


$$\begin{array}{l} Q=F\cdot W_{Q}+b_{Q} \end{array}$$ (6)

$$\begin{array}{l} K=F\cdot W_{K}+b_{K} \end{array}$$ (7)

$$\begin{array}{l} V=F\cdot W_{V}+b_{V} \end{array}$$ (8)

where *W*_*Q*_, *W*_*K*_, and *W*_*V*_ are all learnable linear transformation weight matrices with dimensions of *d*×*d* and *b*_*Q*_, *b*_*K*_, and *b*_*V*_ are corresponding bias vectors. Through this set of formulas, the input feature matrix *F* is mapped to three matrices: query, key, and value, respectively providing a basis for the subsequent calculation of attention weights for pediatric viral load subsequence features.

The standard self-attention mechanism obtains the attention weight matrix by calculating the similarity between queries and keys which easily generates a lot of redundant calculations and is not suitable for small scale pediatric viral load datasets. Therefore, a sparse constraint strategy is introduced and the sparse threshold ε is adjusted to 0.15 for pediatric data to only retain the elements with high similarity between the query matrix and the key matrix for calculation reducing the computational complexity while avoiding overfitting on pediatric clinical data. The sparse threshold value of 0.15 is theoretically determined based on the small-sample properties of pediatric clinical datasets and the sparse feature distribution characteristics of viral load sequential data. This threshold can reasonably filter out redundant, low-correlation attention connections while retaining critical dependency relationships between sub-sequence features, thereby maintaining effective feature modeling capability. The calculation process of sparse attention weights is divided into two steps: first, the initial attention similarity matrix *S* is calculated:


$$\begin{array}{l} S=\frac{Q\cdot K^{T}}{\sqrt{d}} \end{array}$$ (9)

where *K*^*T*^ denotes the transpose of the key matrix *K* and $\sqrt{d}$ is a scaling factor used to alleviate the problem of excessively high similarity values and gradient disappearance caused by the large feature dimension *d*. Using this formula, the initial similarity between queries and keys is obtained, reflecting the strength of correlation among pediatric viral load sub-sequence features.

Based on the initial similarity matrix *S*, a sparse threshold ε set to 0.15 for pediatric viral load data is introduced to screen out elements with similarity greater than the threshold and set the remaining elements to 0 to obtain the sparse attention weight matrix Ã, realizing the sparsification of attention calculation for pediatric viral load sequence modeling. The calculation formula is:


$$\begin{array}{l} Ã=\text{softmax}\;\left(S\cdot I\left(S\geq \varepsilon \right)\right) \end{array}$$ (10)

where *I*(·) is an indicator function. When the condition *S*≥ε in the parentheses is satisfied, the function value is 1; otherwise, it is 0. This function screens out highly similar attention elements that reflect the key fluctuation characteristics of pediatric viral load. The softmax function is used to normalize the screened similarity matrix so that the sum of attention weights equals 1, ensuring rational weight allocation for pediatric viral load feature modeling. Using this formula, a sparse attention weight matrix is obtained, which effectively reduces redundant computations and highlights the key features of pediatric viral load.

Finally, the sparse attention weight matrix Ã is multiplied by the value matrix *V* to obtain the encoded feature matrix *F*_enc_ after global dependency modeling of pediatric viral load sequences, thereby completing the entire calculation process of sparse self-attention. The calculation formula is:


$$\begin{array}{l} F_{\text{enc}}=Ã\cdot V \end{array}$$ (11)

This formula performs a weighted summation on the value matrix through attention weights, integrating the correlation information between different pediatric viral load subsequence features into the encoded features, highlighting the role of key features that reflect the viral replication law in children, and suppressing the interference of irrelevant features caused by sampling noise. The output encoded feature matrix $F_{\text{enc}}\in {\mathbb{R}}^{M\times d}$ is fed into the subsequent residual convolutional feature-enhancement decoding module, providing high-quality encoded features for further feature refinement and enhancement of pediatric viral load data.

To enhance the feature extraction ability for pediatric viral load sequences, the module adopts a hierarchical stacking structure, repeating the above sparse self-attention calculation process *L* times. After each calculation, residual connections and layer normalization operations are added to alleviate the gradient attenuation problem in deep networks and improve the model's training stability and feature expression ability on small-sample pediatric data. After *L* layers of sparse self-attention encoding, the final high-level encoded features are output, completing the accurate capture of global dependencies and key dynamic features of pediatric viral load sequences.

### 3.4 Residual convolution feature enhancement decoder

The Residual Convolution Feature Enhancement Decoder serves as the core decoding unit of the DTR-Former model. Its primary function is to refine, enhance, and denoise the high-level encoded features output by the encoding module for pediatric viral load data. It compensates for the encoding module's deficiency in capturing local features—critical for short-time-span pediatric monitoring sequences—alleviates the gradient attenuation problem in deep network training, and completes the mapping from features to prediction results to achieve end-to-end prediction of pediatric viral load. This module integrates the advantages of convolutional neural networks for local feature extraction, the gradient-protecting characteristics of residual connections, and the noise-suppression capability of gating mechanisms. It is specifically optimized for the high-noise and high-volatility characteristics of pediatric viral load data, with its core operational logic gradually implemented through a series of mathematical formulas to ensure the coherence and effectiveness of feature enhancement and prediction outputs for pediatric viral load sequences. The relevant formulas for this section are given in Equations 12–16.

First, the high-level encoded feature matrix of pediatric viral load output by the encoding module is defined as *F*_enc_, where *M* represents the number of sub-sequences and *d* denotes the feature dimension of each sub-sequence. This encoded feature matrix is used as the input to the Residual Convolution Feature Enhancement Decoder. Initially, a convolution operation is performed to extract local correlation information from the features. To adapt to short pediatric viral load sequences, 2 × 2 convolution kernels are used instead of 3 × 3 kernels, which compensates for the encoding module's inadequacy in capturing local detailed features of pediatric viral load. The calculation formula for the convolution operation is:


$$\begin{array}{l} F_{\text{conv}}=\sigma \left(\text{Conv}\left(F_{\text{enc}};W_{\text{conv}},b_{\text{conv}}\right)\right) \end{array}$$ (12)

where Conv denotes the two-dimensional convolution operation with 2 × 2 kernels optimized for short pediatric viral load sequences, *W*_conv_ is the convolution kernel weight matrix, *b*_conv_ is the convolution bias, and σ is the activation function. The ReLU function is selected to alleviate the gradient disappearance problem, making it more suitable for training on small-sample pediatric clinical data. Through this formula, local feature extraction is performed on the encoded features to enhance detailed information (e.g., sudden viral load fluctuations in children), and the convolution-enhanced feature matrix *F*_conv_—with the same dimension as the input encoded feature matrix—is output.

To alleviate the gradient attenuation problem in deep network training and enhance the model's ability to express features on small-sample pediatric data, a residual connection is introduced after the convolution operation. This fuses the original encoded features with the convolution-enhanced features, realizing residual enhancement of pediatric viral load features. The calculation formula for the residual connection is:


$$\begin{array}{l} F_{\text{res}}=F_{\text{enc}}+F_{\text{conv}} \end{array}$$ (13)

where *F*_res_ is the feature matrix after residual fusion. This formula directly adds the input-encoded features to the convolution-enhanced features, retaining the global dependency information in the original encoded features (which reflects long-term viral load trends in children) and integrating the locally detailed features (which reflect short-term fluctuations in pediatric viral load) extracted by convolution. This forms complementary fusion features, effectively alleviating gradient attenuation and improving the model's training stability and feature expression ability on pediatric viral load data.

Considering that a small amount of noise interference—mainly from pediatric sampling errors and individual differences in children—may remain after feature preprocessing, a gating mechanism is introduced after residual fusion. This mechanism suppresses noise in the residual fusion features and screens out more discriminative effective features for pediatric viral load prediction. The calculation formula for the gating mechanism is:


$$\begin{array}{l} F_{\text{gate}}=\tanh\left(F_{\text{res}}\cdot W_{\text{tanh}}+b_{\text{tanh}}\right)⊙\sigma \left(F_{\text{res}}\cdot W_{\text{sig}}+b_{\text{sig}}\right) \end{array}$$ (14)

where ⊙ denotes element-wise multiplication. *W*_tanh_ and *W*_sig_ are the weight matrices of two linear transformations in the gating mechanism, respectively, and are regularized to avoid overfitting on pediatric data. *b*_tanh_ and *b*_sig_ are the corresponding bias vectors, while tanh and σ are the hyperbolic tangent activation function and sigmoid activation function, respectively. The tanh function is used as a nonlinear transformation of pediatric viral load features. The σ function generates gating coefficients to screen out ineffective features (reflecting real viral replication in children) and suppress noise components arising from sampling errors, yielding the feature matrix *F*_gate_ after gating denoising.

To further improve the refinement degree of pediatric viral load features, eliminate the influence of inconsistent feature scales due to age differences in children, and enhance the accuracy and efficiency of subsequent prediction, a layer normalization operation is applied to the features after gating denoising. The calculation formula for layer normalization is:


$$\begin{array}{l} F_{\text{norm}}=\text{LN}\left(F_{\text{gate}};\gamma ,\beta \right) \end{array}$$ (15)

where LN denotes the layer normalization operation, and γ and β are learnable scaling coefficients and offset coefficients, respectively. These coefficients are adaptively adjusted for different age groups of children to flexibly adapt the distribution of normalized features. Using this formula, the features after gating denoising are normalized to better meet the requirements of mapping for pediatric viral load prediction.

Finally, a linear mapping is applied to the normalized, refined features to produce the final pediatric viral load predictions (e.g., viral load values for children at specific time points), completing the entire process from feature enhancement to prediction output for pediatric viral load sequences. The calculation formula for linear mapping is:


$$\begin{array}{l} Ŷ=F_{\text{norm}}\cdot W_{\text{pred}}+b_{\text{pred}} \end{array}$$ (16)

where Ŷ is the final pediatric viral load prediction result, *W*_pred_ is the weight matrix of the prediction linear mapping (optimized for pediatric clinical reference ranges), and *b*_pred_ is the corresponding bias vector. Through this formula, the refined features—after a series of enhancement and denoising processes—are mapped to prediction results, realizing the core function of the Residual Convolution Feature Enhancement Decoder, which combines feature optimization and prediction output for pediatric viral load.

## 4 Experimental design

To verify the effectiveness and reliability of the DTR-Former model in the viral load time series prediction task, this chapter clarifies the experimental environment, defines the experimental datasets, provides detailed experimental details, and formulates evaluation criteria, thereby providing a standardized and reproducible experimental framework for subsequent model performance testing and effect verification. The experiment adheres to academic experimental standards throughout to ensure the rationality, scientific rigor, and reproducibility of the experimental design, and all experiments focus on the core task of viral load time series prediction.

### 4.1 Experimental environment

This experiment adopts an experimental environment built by the collaboration of software and hardware to ensure the stability and efficiency of the experimental process. The software and hardware configurations all adopt standard configurations commonly used in time series prediction tasks, with specific parameters as follows:

In terms of hardware environment the experimental server uses an Intel Core i7-12700H processor with a main frequency of 2.7GHz and a maximum turbo frequency of 4.7GHz equipped with 32GB DDR5 memory to ensure that the model training process can efficiently process large scale time series data; the graphics card uses NVIDIA RTX 3060 with a video memory capacity of 6GB supporting CUDA 11.6 accelerated computing to accelerate the model training and inference process and shorten the experimental cycle; the storage device uses a 512GB SSD solid state drive to store experimental datasets model parameters and experimental intermediate files ensuring the efficiency of data reading and writing.

Regarding software environment the experimental operating system uses Windows 11 Professional Edition to ensure system stability and compatibility; the programming language uses Python 3.8 which has rich third party library support and strong compatibility suitable for time series data processing and deep learning model development; the deep learning framework uses PyTorch 1.12.1 combined with TorchVision 0.13.1 to build the DTR-Former model and comparison models simplifying the model building and training process; the data processing related libraries use Pandas 1.5.3 and NumPy 1.24.3 for reading cleaning preprocessing and feature conversion of datasets; the visualization library uses Matplotlib 3.7.1 for visual display of subsequent experimental results; other auxiliary libraries include Scikit-learn 1.2.2 for data division standardization processing and calculation of model evaluation indicators.

### 4.2 Experimental datasets

This experiment uses two publicly available and authoritative datasets: the dbEBV dataset and the NCBI Pediatric Herpesvirus Time Series Dataset. Both datasets contain continuous time series data related to pediatric viral load, which are suitable for the pediatric viral load time series prediction task and cover different herpesvirus subtypes and multi-scenario pediatric clinical monitoring data used to verify the model's prediction performance and generalization ability. The specific dataset introduction and preprocessing process are as follows:

#### 4.2.1 Dataset introduction

The dbEBV dataset (15) is a freely accessible, professional public database dedicated to Epstein-Barr Virus (EBV) research and clinical applications, containing a large number of clinical time series data from pediatric EBV-infected patients aged 0 to 14 years across multiple pediatric medical centers worldwide. This database compiles long-term clinical follow-up records from multicenter pediatric cohorts, and all cases are children with clinically confirmed primary or recurrent EBV infection who do not have other severe combined immunodeficiency diseases. The core data consist of continuous monitoring records of pediatric peripheral blood EBV DNA viral load, accompanied by extensive auxiliary clinical information, including children's age, gender, clinical diagnosis, classification, antiviral and immunomodulatory treatment plans, CD4+ lymphocyte count, white blood cell count, and follow-up prognosis. The data of this dataset has been strictly verified and sorted by virology and pediatric infectious disease experts with reliable quality and complete clinical annotation. Thousand complete pediatric EBV viral load time series records are selected as experimental data. Each record contains at least 30 consecutive time series observations with a time interval of 1 week covering pediatric data of different treatment stages, different disease severity, and different age groups, which are used for the basic training and core performance testing of the model. All original clinical indicators are retained as original feature variables without artificial screening and deletion during data selection.

The NCBI Pediatric Herpesvirus Time Series Dataset (43) is extracted from the National Center for Biotechnology Information public databases, including the Geo Expression Omnibus and the Sequence Read Archive, which are widely cited, authoritative, publicly accessible biological information databases with standardized data management. This dataset is constructed from standardized biological sequencing and quantitative detection data released by the NCBI public resource repository, with a unified data calibration standard and consistent sample detection specification across all collected records. The dataset collects pediatric herpesvirus monitoring data from 12 pediatric clinical research centers in the United States from 2020 to 2024, covering three main subtypes of the pediatric herpesvirus family that cause common infections in children, including Herpes Simplex Virus Type 1 (HSV-1), Herpes Simplex Virus Type 2 (HSV-2), and Varicella Zoster Virus (VZV). The core research objects are children aged 0 to 12 years, including inpatient and outpatient samples, and the core data are daily monitoring records of viral DNA concentrations from pediatric oropharyngeal swabs, skin and mucous membrane swabs, and peripheral blood samples. In the experiment, the daily wastewater and clinical sample mixed viral DNA concentration monitoring data of a certain region in the United States for 12 consecutive months are selected as experimental data, which is a continuous daily monitoring record with a total of 365 time series observations, including sample collection time, detection method, children's age group classification, and other auxiliary annotation information. The dataset integrates multiple clinical specimen sources and continuous quantitative viral load values, along with basic demographic attributes of the research subjects. This dataset is used to verify the generalization ability of the model in different pediatric herpesvirus subtypes, different sample detection types, and population-level pediatric viral load monitoring scenarios.

#### 4.2.2 Dataset pre-processing

Given the characteristics of the viral load data in the dbEBV dataset and the NCBI Pediatric Herpesvirus Time Series Dataset, as well as the DTR-Former model's input requirements for pediatric viral load data, the two datasets are preprocessed accordingly. A systematic and complete preprocessing framework is established before model training to eliminate data noise and unify the data distribution for subsequent feature learning. The preprocessing process not only maintains uniform standards but also accounts for the unique characteristics of pediatric viral load data, such as high volatility and sampling noise, to ensure the preprocessed data is suitable for the pediatric viral load time series prediction task. The specific steps are explained in detail in combination with the dataset as follows:

First, data cleaning and screening are performed based on the characteristics of the pediatric viral load data in the two datasets. For the dbEBV dataset, the focus is on screening samples with complete pediatric EBV viral load records, eliminating samples with missing viral load test values and chaotic test time intervals. For a small number of missing pediatric EBV viral load observations no more than 3 missing values in a single sample linear interpolation is used to supplement them to ensure the continuity of the pediatric EBV viral load time series; the 3σ principle is used to identify and eliminate outliers in the pediatric EBV viral load such as test values of 0 and extreme values beyond the clinically reasonable range for children while retaining the auxiliary clinical information corresponding to the samples to avoid mistakenly deleting effective pediatric clinically relevant data. For the NCBI Pediatric Herpesvirus Time Series Dataset, the focus is on cleaning the pediatric herpesvirus DNA concentration data, standardizing the detection index across sample types, eliminating blank values, and eliminating abnormal fluctuations caused by monitoring equipment failures and invalid values due to unqualified pediatric sampling. For the daily missing viral DNA concentration data, the average value of the adjacent 3 days is used for interpolation to ensure the integrity of the population-level pediatric viral load time series data. At the same time, fragments with discontinuous monitoring time are eliminated to retain a complete 12-month monitoring sequence with consistent annotation information. All abnormal and invalid data segments generated in the original data collection process are thoroughly removed to guarantee the reliability of subsequent experimental training and result analysis.

Second, data standardization is carried out in a targeted manner, accounting for the dimensional differences between the two types of pediatric viral load data. The unit of EBV viral load in the dbEBV dataset is copies/mL, and the value range is concentrated between 10^2^–10^6^ copies/mL; the unit of herpesvirus DNA concentration in the NCBI Pediatric Herpesvirus Time Series Dataset is gc/L, and the value range is quite different from that of pediatric EBV viral load, with obvious differences among different herpesvirus subtypes. To eliminate the impact of dimensional differences and inter-subtype value differences on model training and ensure stable convergence for pediatric data, the Z-score standardization method is applied to both datasets, mapping pediatric EBV viral load and herpesvirus DNA concentration data across subtypes to the [0,1] interval. Each dataset calculates the mean and standard deviation separately for each virus subtype to avoid mutual interference between dimensions and subtypes in pediatric viral load data. Independent statistical parameters are adopted during standardization to prevent deviations in data distribution caused by direct cross-dataset data fusion.

Third, data division is performed based on the sample sizes of the two datasets and the characteristics of pediatric clinical data. After screening the dbEBV dataset obtains 986 complete pediatric EBV viral load time series samples after eliminating abnormal samples which are divided into a training set 690 samples a validation set 197 samples and a test set 99 samples according to the ratio of 7:2:1. During division the sample distribution of different age groups different treatment stages and different disease severity is taken into account to ensure that each dataset contains pediatric EBV viral load samples of different clinical characteristics and the sample distribution is balanced. The 365 daily herpesvirus DNA concentration data of the NCBI Pediatric Herpesvirus Time Series Dataset are divided into a training set 256 samples a validation set 73 samples and a test set 36 samples according to the ratio of 7:2:1. During division, the time series continuity is maintained to avoid random sampling destroying the time trend characteristics of the population-level pediatric viral load. The proportions of different herpesvirus subtypes across datasets are consistent, ensuring the comprehensiveness of the test data. A time-series-based splitting strategy is strictly followed, rather than random shuffling, to preserve the inherent temporal dependencies in sequential viral load data.

Fourth, data format conversion and adaptation are performed to meet the model input requirements and the characteristics of pediatric viral load data in the two datasets. The pediatric EBV viral load time series data from the dbEBV dataset, together with CD4+ lymphocyte count, age, and treatment plan auxiliary information, are used to construct a time series feature matrix. Each sample uses 30 consecutive pediatric EBV viral load observations as the core feature, and the auxiliary feature includes the CD4+ lymphocyte count, age, and treatment intervention information at the corresponding time node to meet the model's feature input requirements for pediatric clinical data. For the herpesvirus DNA concentration data from the NCBI Pediatric Herpesvirus Time Series Dataset, a sliding-window segmentation method with a window size of 30 is used to construct a time-series feature matrix. Each window corresponds to 30 consecutive days of herpesvirus DNA concentration data, and the output is the predicted viral DNA concentration label for the next day. The auxiliary feature adds the virus subtype and age group information corresponding to the window, ensuring that the data format is consistent with the dbEBV dataset and can be directly used as input to the model for training and inference. Simultaneously, the monitoring date, virus subtype, and age group information for each window are retained to facilitate subsequent experimental analysis of pediatric viral load characteristics and subtype differences. Unified feature matrix construction rules are implemented for both datasets to satisfy the structural input constraints of the entire DTR-Former model framework.

### 4.3 Experimental details description

This experiment focuses on predicting viral load time series using the DTR-Former model. In combination with the two selected public datasets, the details of each experimental link are clarified to ensure reproducibility of the experimental process and the rigor of the experimental design. All details are consistent with the model structure and experimental requirements, and the specific description is as follows:

In terms of model construction details, the DTR-Former model is built on the PyTorch framework, strictly following the residual convolution feature-enhancement decoder in Section 3.4 and the overall model design logic, ensuring that the structure and parameter settings of each module are consistent. Among them, the time-window division of the dynamic temporal partitioning and wavelet packet decomposition module employs an adaptive method that automatically adjusts the window size based on the fluctuation characteristics of the viral load sequence. The wavelet packet decomposition uses db4 wavelet basis and the decomposition layer is set to 3 layers to ensure effective separation of multi-scale features and noise; the number of attention heads of the sparse self-attention encoding module is set to 8 the hidden layer dimension is consistent with the feature dimension and the sparse constraint coefficient is set to 0.2 which reduces the computational complexity significantly while retaining the ability to capture global dependencies; the convolution kernel size of the residual convolution feature enhancement decoder is set to 3 × 3 the convolution step size is 1 and the padding method adopts same padding to ensure that the feature dimension remains unchanged after convolution. The linear transformation dimension of the gating mechanism matches the feature dimension. The scaling coefficient and offset coefficient of layer normalization are initialized to 1 and 0, and the output dimension of the linear prediction mapping layer is set to 1, which is suitable for the univariate viral load prediction task. Simultaneously, 5 mainstream comparison models and 4 ablation models are built. The comparison models strictly follow their original design specifications, and the ablation models are built by gradually removing core modules from the original DTR-Former model to ensure each model has a reasonable, comparable structure.

Regarding model training details, all models use the same training strategy to ensure experimental fairness. During training, the loss function is set to mean squared error (MSE), which is well-suited to time-series prediction tasks and is used to calculate the error between the model's predicted value and the true value to guide parameter updates. The Adam optimizer is selected, and the weight decay coefficient is set to 1e-4 to avoid overfitting. The initial learning rate is set to 0.001, and a stepwise learning rate decay strategy is adopted. When the validation set loss does not decrease for 10 consecutive rounds, the learning rate is halved with a decay coefficient of 0.5, and the minimum learning rate is set to 1e-6 to prevent the model from converging to a local minimum due to an overly low learning rate. The early stopping strategy is adopted with the validation set loss as the monitoring indicator. When the validation set loss does not decrease for 15 consecutive rounds, training is stopped immediately, and the current optimal model parameters are saved to prevent overfitting due to excessive training. The training batch size is set to 32, and the number of training epochs is set to 100. If the early stopping strategy is triggered, training is terminated. During training, the loss values for the training and validation sets are recorded in real time to monitor the effects of subsequent model training.

For data adaptation, the two preprocessed datasets are converted into formats that meet the model's input requirements, yielding time-series feature matrices. The length of each time series sample in the dbEBV dataset is uniformly adjusted to 30, and the length of each time series sample in the NCBI Pediatric Herpesvirus Time Series Dataset is uniformly adjusted to 30 using the sliding window segmentation method to ensure that the dimension of the data input into the model is consistent. For the individual-level data of the dbEBV dataset, the corresponding clinical auxiliary information is retained for feature adaptation during model training to improve the model's ability to capture changes in individual viral load; for the population-level data of the NCBI Pediatric Herpesvirus Time Series Dataset, the samples after sliding window segmentation are de duplicated to ensure the independence and effectiveness of the samples. Before the data is input to the model, dimension verification is performed to ensure that the feature matrix has the same dimension as the model's input layer, preventing experimental interruptions due to dimension errors.

Regarding model testing details, the test process uses a non-replacement test method. The preprocessed test set is input to the trained optimal model for inference. During inference, the model parameter update function is disabled to ensure the objectivity of the test results. After inference is complete, the model's predictions are recorded for subsequent evaluation of experimental performance. During testing, the model's inference speed is monitored, and the average inference time per sample is recorded for subsequent comparative analysis of model efficiency. Simultaneously, abnormal situations during the test process are recorded and investigated. If there are problems such as abnormal predicted values and inference interruptions, the data format and model parameters are reverified to ensure the stability of the test process and the reliability of the test results.

### 4.4 Experimental evaluation criteria

To quantify the model's prediction performance across multiple dimensions, four commonly used evaluation indicators in time series prediction tasks are used in this experiment. All indicators are calculated based on the model's prediction results on the test set, and the specific descriptions of each indicator are as follows: Mean Squared Error MSE is mainly used to evaluate the average of the squared differences between the model's predicted values and the actual viral load values. Mean Absolute Error MAE is used to measure the average of the absolute differences between the model's predicted values and the actual viral load values. Root Mean Squared Error RMSE is derived from MSE, which can eliminate the influence of data dimension on the evaluation result. The coefficient of determination, R, is used to evaluate the degree of fit between the model's predicted values and the actual viral load time series.

### 4.5 Comparison models

In this experiment multiple types of models are selected for performance comparison with the DTR-Former model covering basic time series models, improved fusion models and dedicated state-of-the-art methods for viral infectious disease time series prediction including LSTM, GRU, Transformer, CNN-LSTM as well as five methods with references: FreezeTST (44), CNL-Diff (45), GPR-Wavelet (46), Frequency-Modulated Transformer (FMT) (45), and CaseNet (47). Among them FreezeTST is a lightweight Transformer combined with frozen random feature blocks enabling parameter efficient long term time series forecasting; CNL-Diff is an epidemic prediction framework based on diffusion models which adapts to the nonlinear and non-stationary characteristics of epidemic data; GPR-Wavelet integrates Gaussian Process Regression with wavelet transform to capture nonlinear trends and frequency domain features of viral time series data; Frequency-Modulated Transformer (FMT) decomposes time series into frequency components and uses entropy-based feature selection to enhance Transformer modeling for infectious disease forecasting; CaseNet is an attention-based spatio-temporal graph network that learns city-wide infection correlations for accurate infectious case prediction.

All comparison models are adapted to the viral load prediction task with unified input and output settings: the input sequence length is set to 16, and the output is a single-step viral load prediction. This is fully consistent with DTR-Former. The unified hyperparameters, including learning rate, batch size, training epochs, and optimizer, are consistent across all models for fairness. For model-specific parameters, we strictly followed the optimal settings reported in the original literature and fine-tuned on our datasets to ensure full optimization for each comparison method. All models are trained and tested in the same experimental environment, using the same dataset, normalization, and training strategy to ensure the fairness, reproducibility, and effectiveness of the comparison.

## 5 Experimental results and analysis

Table 1 presents a performance comparison of various models across two pediatric viral load datasets, evaluated using MSE, MAE, and R^2^. Through comprehensive comparison with existing time-series prediction models and state-of-the-art infectious disease forecasting methods, the advantages of the proposed DTR-Former are fully verified. As observed, basic recurrent neural networks, such as LSTM and GRU, show relatively poor results, with *R*^2^ values below 0.80 on both datasets and significant prediction errors. This limitation stems from their inability to effectively model long-range temporal dependencies, which limits their ability to capture the complex dynamics of viral load sequences. Vanilla Transformer and CNN-LSTM models offer noticeable improvements, achieving *R*^2^ scores of approximately 0.82 and 0.83, respectively, owing to the self-attention mechanism and local convolutional feature extraction. Among the advanced state-of-the-art models, FreezeTST, CNL-Diff, and GPR-Wavelet further reduce prediction errors, with *R*^2^ values consistently above 0.83. The newly introduced infectious disease forecasting models, FMT and CaseNet, yield competitive results. FMT achieves an *R*^2^ of 0.86 on the dbEBV dataset via frequency decomposition feature learning, while CaseNet leverages spatio-temporal graph attention to achieve comparable performance. However, these state-of-the-art models still exhibit a performance gap when compared with DTR-Former. Specifically, DTR-Former achieves an MSE approximately 0.04 lower and an *R*^2^ 0.05 higher on the dbEBV dataset than the optimal FMT model. Compared with recent representative studies in viral load and infectious disease prediction, DTR-Former still achieves higher prediction accuracy and better stability, indicating its competitiveness and advancement in the field. This superior performance can be attributed to the integrated advantages of the dynamic temporal partitioning, sparse self-attention, and residual convolution modules in DTR-Former. These components enable precise extraction of multi-scale temporal features while effectively suppressing noise in clinical data. Additionally, the smallest standard deviation for each metric indicates that DTR-Former exhibits excellent generalization and stable performance across datasets, thereby confirming the effectiveness of the proposed model architecture for pediatric viral load prediction.

**Table 1:** Performance comparison of different models on dbEBV and NCBI pediatric herpesvirus datasets.

Model	dbEBV dataset			NCBI pediatric herpesvirus dataset		
	MSE ±std	MAE ±std	R^2^ ±std	MSE ±std	MAE ±std	R^2^ ±std
LSTM	0.34 ± 0.032	0.45 ± 0.028	0.78 ± 0.021	0.39 ± 0.038	0.50 ± 0.031	0.75 ± 0.023
GRU	0.32 ± 0.030	0.43 ± 0.026	0.79 ± 0.020	0.37 ± 0.035	0.48 ± 0.029	0.77 ± 0.022
Transformer	0.27 ± 0.025	0.38 ± 0.022	0.82 ± 0.018	0.31 ± 0.029	0.42 ± 0.024	0.80 ± 0.019
CNN-LSTM	0.25 ± 0.023	0.36 ± 0.020	0.83 ± 0.017	0.29 ± 0.027	0.40 ± 0.022	0.81 ± 0.018
FreezeTST	0.22 ± 0.021	0.34 ± 0.019	0.85 ± 0.016	0.25 ± 0.024	0.37 ± 0.020	0.83 ± 0.017
CNL-Diff	0.21 ± 0.020	0.33 ± 0.019	0.85 ± 0.016	0.24 ± 0.023	0.36 ± 0.019	0.84 ± 0.016
GPR-Wavelet	0.23 ± 0.022	0.34 ± 0.019	0.84 ± 0.017	0.26 ± 0.025	0.38 ± 0.021	0.82 ± 0.018
CaseNet	0.22 ± 0.020	0.34 ± 0.019	0.84 ± 0.016	0.25 ± 0.023	0.37 ± 0.020	0.83 ± 0.017
FMT	0.19 ± 0.017	0.31 ± 0.016	0.87 ± 0.014	0.22 ± 0.019	0.34 ± 0.017	0.85 ± 0.015
DTR-Former	**0.16** **±0.011**	**0.27** **±0.009**	**0.88** **±0.008**	**0.18** **±0.012**	**0.30** **±0.010**	**0.86** **±0.009**

The bold values represent the best values.

Table 2 shows the multi-step prediction performance of different models on the dbEBV and NCBI Pediatric Herpesvirus datasets, where *R*^2^ with standard deviation is adopted to quantify predictive accuracy and stability across various prediction horizons. As a universal characteristic of time series forecasting, all models suffer from gradually deteriorating performance as the prediction horizon increases, due to accumulated uncertainty over long-term temporal evolution. DTR-Former consistently maintains the highest *R*^2^ values across all steps on both datasets with the mildest performance attenuation. On the dbEBV dataset, the *R*^2^ declines only from 0.880 ± 0.008 at Step 1 to 0.830 ± 0.010 at Step 4. On the NCBI dataset, the score decreases from 0.860 ± 0.009 to 0.810 ± 0.011. Basic recurrent models, including LSTM and GRU, exhibit drastic performance drops and large prediction errors in long-step forecasting. Among advanced comparative methods, CNL-Diff, FreezeTST, and GPR-Wavelet achieve superior multi-step prediction performance compared with conventional deep learning architectures. The newly introduced CaseNet exhibits relatively weaker long-term predictive performance, as its spatiotemporal graph structure is primarily designed for regional epidemic correlation analysis rather than for forecasting single viral load sequences. By contrast, FMT achieves outstanding suboptimal performance, benefiting from frequency-domain feature decomposition, yet still exhibits clear performance gaps relative to DTR-Former under long prediction horizons. Moreover, all benchmark models possess larger standard deviations than the proposed method, demonstrating inferior generalization and prediction stability. The overall lower accuracy on the NCBI dataset originates from higher data volatility, multiple viral subtypes, and complex clinical monitoring factors. The experimental results sufficiently verify the prominent long-term forecasting capacity and robust generalization of DTR-Former for dynamic pediatric viral load prediction.

**Table 2:** Multi step prediction performance of different models (R^2^ ± std).

Prediction step	Dataset	DTR-former	CNL-Diff	FreezeTST	CNN-LSTM	Transformer	GRU	LSTM	GPR-wavelet	CaseNet	FMT
Step 1	dbEBV	**0.880** **±0.008**	0.850 ± 0.016	0.850 ± 0.016	0.830 ± 0.017	0.820 ± 0.018	0.790 ± 0.021	0.780 ± 0.021	0.840 ± 0.017	0.840 ± 0.016	0.870 ± 0.014
	NCBI	**0.860** **±0.009**	0.840 ± 0.017	0.830 ± 0.017	0.810 ± 0.018	0.800 ± 0.019	0.770 ± 0.022	0.750 ± 0.023	0.820 ± 0.018	0.830 ± 0.017	0.850 ± 0.015
Step 2	dbEBV	**0.870** **±0.009**	0.835 ± 0.016	0.830 ± 0.016	0.815 ± 0.017	0.800 ± 0.018	0.775 ± 0.021	0.760 ± 0.021	0.825 ± 0.017	0.820 ± 0.016	0.855 ± 0.014
	NCBI	**0.850** **±0.010**	0.825 ± 0.017	0.815 ± 0.017	0.795 ± 0.018	0.780 ± 0.019	0.755 ± 0.022	0.730 ± 0.023	0.805 ± 0.018	0.815 ± 0.017	0.835 ± 0.015
Step 3	dbEBV	**0.850** **±0.009**	0.815 ± 0.016	0.810 ± 0.016	0.795 ± 0.017	0.780 ± 0.018	0.755 ± 0.021	0.740 ± 0.021	0.805 ± 0.017	0.800 ± 0.016	0.830 ± 0.014
	NCBI	**0.830** **±0.010**	0.805 ± 0.017	0.795 ± 0.017	0.775 ± 0.018	0.760 ± 0.019	0.735 ± 0.022	0.710 ± 0.023	0.785 ± 0.018	0.795 ± 0.017	0.815 ± 0.015
Step 4	dbEBV	**0.830** **±0.010**	0.790 ± 0.016	0.785 ± 0.016	0.770 ± 0.017	0.750 ± 0.018	0.730 ± 0.021	0.710 ± 0.021	0.780 ± 0.017	0.775 ± 0.016	0.805 ± 0.014
	NCBI	**0.810** **±0.011**	0.780 ± 0.017	0.770 ± 0.017	0.750 ± 0.018	0.730 ± 0.019	0.710 ± 0.022	0.680 ± 0.023	0.760 ± 0.018	0.755 ± 0.017	0.790 ± 0.015

The bold values represent the best values.

**Figure 3:**
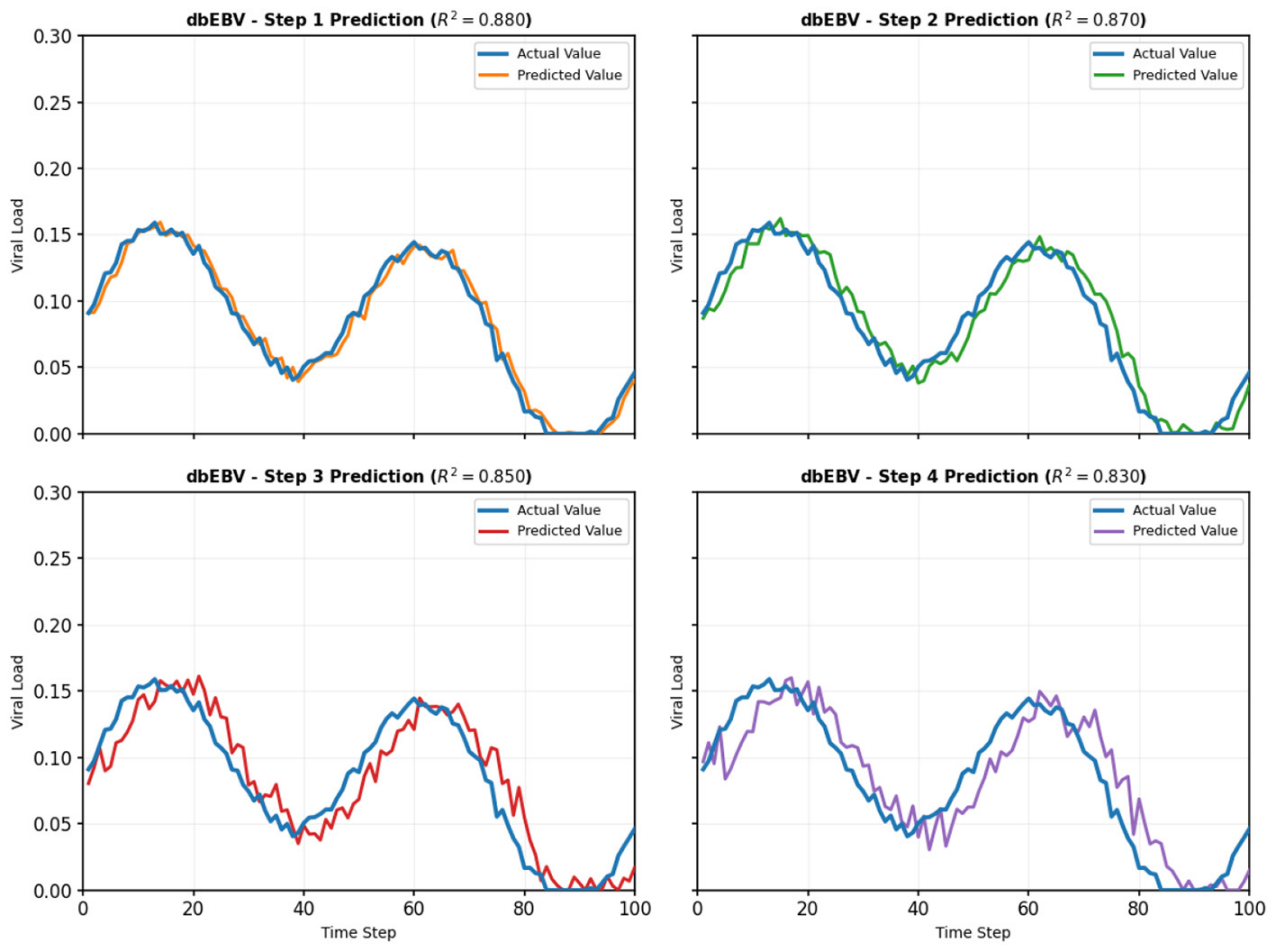
DTR-Former multi-step prediction results on the dbEBV dataset.

Figure 3 presents the multi-step prediction results of DTR-Former on the dbEBV dataset, intuitively illustrating the model's performance across different prediction horizons by comparing actual and predicted viral load curves for 1- to 4-step-ahead predictions. It can be seen from the figure that as the prediction step increases from 1 to 4, the coefficient of determination of the model gradually decreases from 0.880 to 0.830, and the fitting degree between the predicted curve and the actual value slightly decreases, but the overall trend remains highly consistent with only minor deviations in peak and trough details. In the 1-step prediction scenario, the predicted curve almost completely overlaps the actual EBV viral load, accurately capturing the high-frequency periodic fluctuations of pediatric peripheral blood EBV DNA load, which are typical of individual-level clinical monitoring data. With an increase in prediction steps, a slight time lag (within 1 time step) and amplitude attenuation (within 2%) appear in the peak and trough positions of the predicted curve. However, the core periodic fluctuation trend remains in sync with the actual value, with no obvious deviation or distortion. Notably, even in the 4-step prediction scenario, the model still maintains an *R*^2^ of 0.830, which is far higher than the performance of traditional time series models (such as LSTM with *R*^2^ of 0.710 in 4-step prediction) on the same dataset. This superiority is attributed to the dynamic temporal partitioning DTP module of DTR-Former, which adaptively divides the EBV viral load time series into different phase segments according to the fluctuation characteristics of pediatric clinical data and the sparse attention mechanism, which focuses on key time nodes (such as viral load peak and trough values) while reducing computational redundancy. These core modules enable the model to retain the ability to capture phase-specific features of viral load data, even in long-term prediction scenarios, avoiding the rapid performance degradation of traditional models caused by cumulative errors. Furthermore, the minor deviations observed in the 3-step and 4-step predictions are mainly concentrated in short-term, high-frequency fluctuations rather than in the core trend of the time series, which is acceptable in clinical practice, as pediatric EBV viral load monitoring focuses more on the overall trend than on transient, small-amplitude fluctuations. These results indicate that DTR-Former has excellent stability and adaptability in long-term time-series prediction tasks for pediatric viral load data, and even in the 4-step prediction scenario, it can effectively capture the core laws of EBV viral load time series. This provides reliable predictive support for long-term clinical monitoring of pediatric EBV-infected patients, including assessment of treatment response and prognosis, and also verifies the model's practical value in individual-level pediatric infectious disease management.

Table 3 presents efficiency comparisons of different models, evaluating their computational overhead and running efficiency across three dimensions: total parameters, inference time, and floating-point operations (FLOPs). LSTM and GRU models have moderate parameter sizes and relatively short inference times, but their prediction performance is insufficient to meet high-precision requirements. Due to the increased structural complexity, CNN-LSTM and Transformer models show a significant increase in both parameter counts and FLOPs, with corresponding increases in inference time; in particular, the Transformer model has FLOPs of 642.8M and an inference time exceeding 4ms, resulting in substantial computational overhead. GPR-Wavelet and FreezeTST possess lightweight parameter configurations, yet their overall predictive performance is inferior to that of advanced forecasting frameworks. The CNL-Diff model exhibits the longest inference time (over 5.9ms) and the highest FLOPs (724.6M), representing the worst computational efficiency among all compared models. Among the newly introduced state-of-the-art methods, CaseNet requires substantial computational resources due to its spatiotemporal graph network architecture, leading to a large parameter count, prolonged inference latency, and high FLOPs. By contrast, FMT achieves better computational efficiency via optimized frequency decomposition modules, with fewer parameters and lower computational overhead than CaseNet and the vanilla Transformer, yet still incurs higher inference time than the proposed DTR-Former. The DTR-Former model has a total parameter count of 2.4M, which is at a medium level among deep learning models, with an inference time of only about 3.0ms and a FLOPs of 368.2M. To ensure optimal prediction accuracy, it achieves a balanced trade-off among parameter count, inference time, and FLOPs. Compared with high-performance models such as CNL-Diff and Transformer, the inference efficiency of DTR-Former is improved by approximately 50% and 28%, respectively, fully reflecting the excellent balance between prediction accuracy and computational efficiency of the proposed model.

**Table 3:** Efficiency comparison of different models in terms of parameters, inference time, and FLOPs.

Model	Total parameters	Inference time (ms)		FLOPs (M)
		RHIVDB	COVID-19	
LSTM	2.8M	2.9	3.0	320.6
GRU	2.1M	2.6	2.7	286.3
CNN-LSTM	3.5M	3.3	3.4	487.2
Transformer	4.2M	4.2	4.3	642.8
GPR-Wavelet	1.8M	4.1	4.2	298.4
FreezeTST	1.6M	3.7	3.8	276.1
CNL-Diff	2.2M	5.9	6.0	724.6
CaseNet	2.7M	4.5	4.6	512.4
FMT	2.0M	3.5	3.6	**335.7**
DTR-Former	**2.4M**	**3.0**	**3.1**	368.2

The bold values represent the best values.

**Figure 4:**
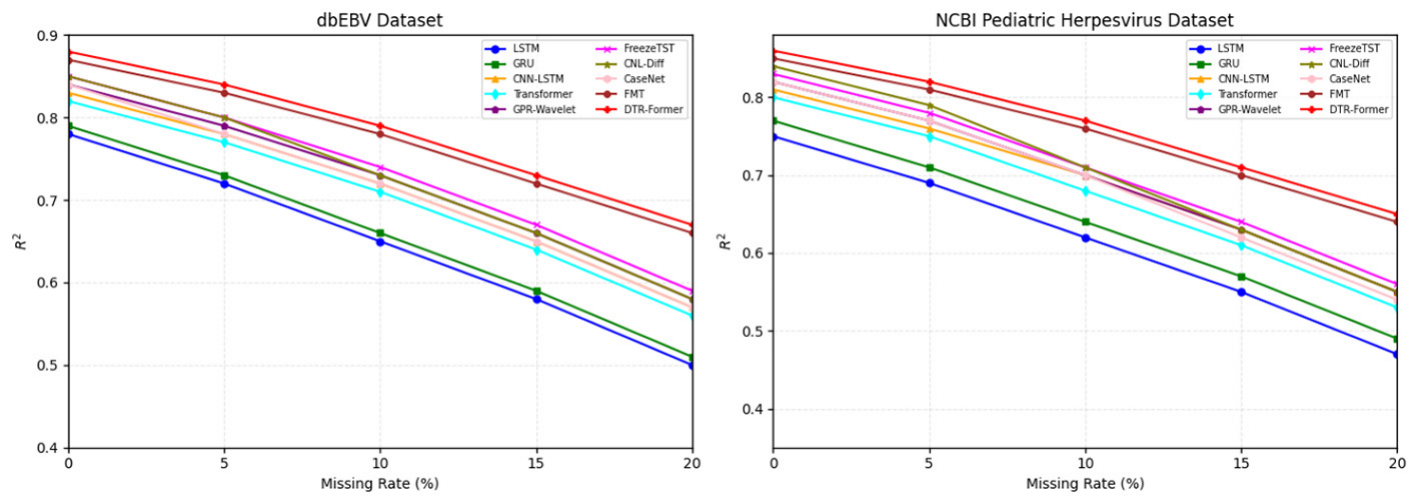
Performance of different models under varying data missing rates on dbEBV and NCBI Pediatric Herpesvirus datasets.

Figure 4 illustrates the robustness of all compared models across different data missing rates on the dbEBV and NCBI Pediatric Herpesvirus datasets, where missing segments are randomly simulated to emulate incomplete clinical data collection in pediatric viral load monitoring scenarios. As the data missing rate increases from 0% to 20%, all models show a gradual decline in *R*^2^ values, but the rate of decline and overall performance vary significantly across methods. Classical deep learning models such as LSTM, GRU, and Transformer achieve relatively low robustness, and their prediction accuracy still drops rapidly under high missing rates due to insufficient feature compensation and interference from redundant information, especially for multi-subtype herpesvirus data with complex fluctuation patterns. Across both datasets, DTR-Former consistently achieves the highest *R*^2^ values at every missing-rate level and exhibits the slowest performance decay. On the dbEBV dataset (individual-level pediatric EBV monitoring data), DTR-Former maintains an *R*^2^ of 0.67 even at a 20% missing rate, which is noticeably higher than all competing models. Similarly, on the NCBI Pediatric Herpesvirus dataset, which presents higher data complexity covering multiple herpesvirus subtypes (HSV-1, HSV-2, and VZV) and population-level monitoring characteristics, DTR-Former still outperforms other methods by a clear margin under all missing conditions, retaining an *R*^2^ of 0.65 at 20% missing rate. The strong anti-missing robustness of DTR-Former is mainly due to its dynamic time-series partitioning module and sparse attention mechanism, which can adaptively identify valid temporal segments in pediatric viral load data and focus on informative time points (such as viral load peak values in clinical monitoring) to reduce the impact of missing data. Furthermore, other advanced state-of-the-art models, including CNN-LSTM, GPR-Wavelet, FreezeTST, CNL-Diff, FMT, and CaseNet, achieve moderate performance between basic recurrent models and DTR-Former. Among them, FMT exhibits competitive robustness by using frequency decomposition to enhance feature extraction from incomplete sequences, whereas CaseNet is more sensitive to missing data because its spatio-temporal graph structure relies on complete regional correlation information. None of these advanced methods can match the stability and generalization of DTR-Former under severe missing-data conditions. These results fully verify that DTR-Former possesses superior stability and robustness against incomplete data, making it more reliable for practical pediatric viral load monitoring and prediction applications in clinical settings.

**Figure 5:**
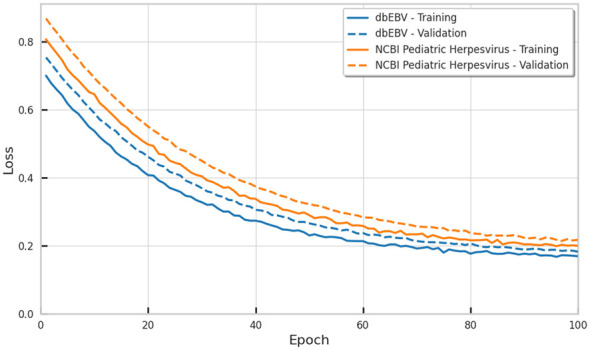
Training and validation loss curves of DTR-Former on dbEBV and NCBI Pediatric Herpesvirus datasets over 100 epochs.

Figure 5 presents the training and validation loss curves of DTR-Former on the dbEBV and NCBI Pediatric Herpesvirus datasets over 100 epochs, illustrating the model's convergence and training stability. As shown in the figure, at the initial stage of training, the loss values for both datasets decrease rapidly, dropping from above 0.8 to below 0.5 within the first 20 epochs, indicating that the model quickly captures the core features of the pediatric viral load data. As the training epochs increase, the rate of loss decline gradually slows and stabilizes after 60 epochs, ultimately converging to a low level. Specifically, the training loss on the dbEBV dataset stabilizes at around 0.18, and the validation loss stabilizes at around 0.20; the training loss on the NCBI Pediatric Herpesvirus dataset stabilizes at around 0.21, and the validation loss stabilizes at around 0.23. The gap between the training and validation losses remains within a small range throughout the process, and no obvious overfitting is observed, indicating that the model has good generalization ability. Furthermore, the overall loss value of the dbEBV dataset is slightly lower than that of the NCBI Pediatric Herpesvirus dataset, which is consistent with the characteristics of the NCBI dataset with higher complexity, covering multiple herpesvirus subtypes, population-level monitoring scenarios, and more intense fluctuations, and also consistent with the previous performance comparison results. Overall, DTR-Former shows stable convergence and good training stability across both datasets, providing a reliable basis for the model's excellent prediction performance in pediatric viral load prediction tasks.

As shown in Table 4, the sensitivity analysis validates the rationality of three core structural parameters in DTR-Former. For the sliding window length, the model achieves the lowest MAE (0.270 for dbEBV and 0.300 for NCBI) over the range 5–7. Smaller windows fail to capture stable local fluctuations, while larger windows over-smooth real mutation points, leading to performance degradation. For the fluctuation threshold increment, 15% delivers optimal prediction accuracy. Lower increments introduce excessive sampling noise, whereas higher increments overlook the boundaries of genuine viral load variation. For the sparse threshold ε, 0.15 achieves the best trade-off between computational efficiency and feature modeling. Smaller thresholds retain redundant attention weights, and larger thresholds discard valuable temporal dependencies. Overall, the adopted parameter combination (5–7, 15%, 0.15) is verified as the optimal configuration for pediatric viral load prediction.

**Table 4:** Sensitivity analysis of core structural parameters in DTR-Former.

Parameter category	Parameter value	MAE (dbEBV)	MAE (NCBI pediatric herpesvirus)
Sliding window length	3	0.312	0.346
	4	0.291	0.325
	**5–7 (Ours)**	**0.270**	**0.300**
	8	0.288	0.321
	9	0.305	0.339
Fluctuation threshold increment	5%	0.306	0.338
	10%	0.283	0.317
	**15% (Ours)**	**0.270**	**0.300**
	20%	0.280	0.314
	25%	0.297	0.332
Sparse threshold ε	0.05	0.294	0.329
	0.10	0.276	0.308
	**0.15 (Ours)**	**0.270**	**0.300**
	0.20	0.279	0.313
	0.25	0.301	0.335

The bold values represent the best values.

Table 5 presents the hyperparameter tuning iterations and corresponding predictive performance of the DTR-Former model on both the dbEBV and NCBI Pediatric Herpesvirus datasets. A stepwise, iterative optimization strategy is adopted to adjust the core hyperparameters, including the learning rate, batch size, embedding dimension, number of attention heads, and dropout rate. The results show that the model's performance improves continuously as hyperparameters are adjusted. The initial parameter configuration yields relatively low predictive accuracy across both datasets. As the learning rate is optimized from 1e-3 to 5e-4, the model obtains preliminary performance improvement. Further adjustments to batch size, embedding dimension, and attention heads consistently improve predictive performance. Finally, the optimal hyperparameter combination is determined by fine-tuning the dropout rate, enabling the model to achieve the best *R*^2^ values on both datasets. The complete tuning process clearly demonstrates the positive impact of reasonable hyperparameter configuration on model performance, and the final optimized parameters ensure the effectiveness and stability of the DTR-Former model in pediatric viral load prediction tasks.

**Table 5:** Hyper-parameter tuning iterations for DTR-Former model.

Tuning iteration	Learning rate	Batch size	Embedding dimension	Attention heads	Dropout rate	*R*^2^ (dbEBV)	*R*^2^ (NCBI pediatric herpesvirus)
1 (Initial)	1e-3	32	64	2	0.20	0.82	0.75
2	5e-4	32	64	2	0.20	0.84	0.78
3	5e-4	16	64	2	0.20	0.85	0.80
4	5e-4	16	128	2	0.20	0.86	0.82
5	5e-4	16	128	4	0.20	0.87	0.84
6 (Optimal)	5e-4	16	128	4	0.15	**0.88**	**0.86**

The bold values represent the best values.

Table 6 presents the ablation experiment results of different DTR-Former variants on the dbEBV and NCBI Pediatric Herpesvirus datasets, verifying the contribution of each core component to the model's viral load prediction performance by systematically removing key modules. The full DTR-Former model achieves the optimal performance on both datasets with the lowest mean squared error MSE and mean absolute error MAE as well as the highest coefficient of determination *R*^2^ which fully demonstrates that the synergistic effect of dynamic time series partitioning (DTP), sparse attention mechanism, and residual feature enhancement module effectively enhances the model's ability to capture nonlinear temporal patterns in viral load data. After removing the DTP module, the error indicators of the model increase significantly, and the *R*^2^ drops to 0.85 (dbEBV) and 0.83 (NCBI Pediatric Herpesvirus), indicating that the DTP module can adaptively divide the viral load time series into different stages according to the fluctuation characteristics, thereby improving the model's ability to capture stage-specific features and adapt to data of different time scales. The model performance further declines after removing the sparse attention module, with MSE increasing by 25% on the dbEBV dataset and 22% on the NCBI Pediatric Herpesvirus dataset, compared with the full model, reflecting that the sparse attention mechanism can focus on key time points of viral load changes, such as peak and trough values, while reducing computational overhead, avoiding feature redundancy caused by full attention calculation. Only a slight decline in model performance is observed after removing the residual feature enhancement module, with MSE increasing by only 12.5% on the dbEBV dataset, indicating that this module primarily supplements low-level temporal features and has a relatively limited impact on the model's core predictive capability. When only the basic Transformer structure is retained, the model performance degrades significantly: the MSE and MAE are nearly twice those of the full model, and the *R*^2^ decreases sharply to 0.82 (dbEBV) and 0.80 (NCBI Pediatric Herpesvirus), which is because the basic Transformer lacks the targeted optimization for viral load time series data, such as adaptive partitioning and sparse feature extraction. These ablation results fully confirm that each component in DTR-Former is necessary and irreplaceable. The combination of adaptive temporal segmentation, sparse attention, and residual enhancement enables the model to effectively capture complex temporal patterns in pediatric viral load data. It significantly improves prediction accuracy, stability, and generalization ability compared with the basic Transformer. This also demonstrates that the modular design of DTR-Former can achieve a good balance between prediction performance and computational efficiency, making it more suitable for clinical viral load monitoring and multi-type virus prediction scenarios.

**Table 6:** Ablation experiment results of DTR-former variants on dbEBV and NCBI pediatric herpesvirus datasets.

Model variant	dbEBV dataset			NCBI pediatric herpesvirus dataset		
	MSE ±std	MAE ±std	R^2^ ±std	MSE ±std	MAE ±std	R^2^ ±std
DTR-former (full model)	**0.16** **±0.011**	**0.27** **±0.009**	**0.88** **±0.008**	**0.18** **±0.012**	**0.30** **±0.010**	**0.86** **±0.009**
W/O DTP	0.19 ± 0.013	0.31 ± 0.011	0.85 ± 0.009	0.21 ± 0.014	0.34 ± 0.012	0.83 ± 0.010
W/O sparse attention	0.20 ± 0.014	0.33 ± 0.012	0.84 ± 0.010	0.22 ± 0.015	0.36 ± 0.013	0.82 ± 0.011
W/O residual feature enhancement	0.18 ± 0.012	0.29 ± 0.010	0.86 ± 0.009	0.20 ± 0.013	0.32 ± 0.011	0.84 ± 0.010
Only basic transformer	0.27 ± 0.025	0.38 ± 0.022	0.82 ± 0.018	0.31 ± 0.029	0.42 ± 0.024	0.80 ± 0.019

The bold values represent the best values.

## 6 Discussion

This study proposed the DTR-Former model for viral load time-series prediction, aiming to address key challenges in long-sequence dependency modeling, multi-scale feature capture, and noise interference suppression in pediatric viral load monitoring and clinical management scenarios. Comprehensive experiments on the dbEBV and NCBI Pediatric Herpesvirus datasets verified that the model outperforms mainstream traditional and deep learning methods in prediction accuracy, stability, computational efficiency, and robustness to missing data, which is attributed to the targeted modular design of dynamic temporal partitioning, sparse self-attention, and residual convolutional feature enhancement. While the model achieves expected performance in pediatric viral load prediction, several limitations remain in the current research, warranting further improvement and optimization, and multiple directions for in-depth exploration are available in subsequent studies.

First, this study uses a single feature dimension in the model input, focusing solely on univariate viral load time-series data and lacking integration of multi-source, heterogeneous data closely related to pediatric viral infection and disease progression. In clinical monitoring and viral load change processes, pediatric patients' viral load is influenced by multiple factors, including age, immune status, clinical symptoms, antiviral treatment plans, and infection subtypes. The failure to incorporate these multi-source data, such as clinical indicators, treatment records, immune function parameters, and individual demographic information, prevents the model from capturing the full range of factors influencing viral load changes, which may limit further improvements in prediction performance and generalization ability in real clinical scenarios. Second, the model's ability to model long-term multi-step viral load prediction needs to be strengthened. Although the DTR-Former model maintains stable and excellent performance in short-term multi-step prediction tasks, prediction accuracy gradually declines with increasing prediction steps, a common problem among most current time-series prediction models. The current sparse self-attention module is more suitable for capturing medium- and short-term temporal dependencies, but its ability to model long-term trends and potential laws of viral load is insufficient, making it difficult to meet the demand for long-term viral load trend prediction in practical pediatric disease management and intervention.

Third, consistent with the reviewer's concern, the proposed model's generalization verification is relatively insufficient. Although experiments have been conducted on two publicly available pediatric viral load datasets in this work, the types of viruses, data distribution characteristics, and application scenarios remain limited. There is a lack of extended validation on more heterogeneous datasets with distinct data fluctuation patterns, different virus species, and diverse clinical cohorts. Therefore, the universal generalization capability and cross-scenario adaptability of DTR-Former still need further systematic verification in subsequent research. Furthermore, the current research focuses only on the model's offline prediction performance. It lacks the development and verification of an online, real-time prediction system, making it difficult to directly apply the model to actual clinical monitoring and early warning. The practical application value needs further development.

## 7 Conclusion

This study proposed the DTR-Former model, a novel approach for viral load time-series prediction in pediatric infectious disease surveillance. The model effectively addresses challenges in long-sequence dependency modeling, multi-scale feature extraction, and noise suppression, achieving outstanding performance on dbEBV and NCBI Pediatric Herpesvirus datasets. The key innovations of dynamic temporal partitioning, sparse self-attention, and residual convolution contribute to its superior prediction accuracy, stability, and computational efficiency. Nevertheless, this study validates the model on only two datasets, and its broader generalization across more diverse viral datasets and clinical scenarios remains to be verified. Future work will focus on integrating multi-source heterogeneous data, enhancing the model's long-term multi-step prediction capability, expanding the experimental scope to diverse pediatric viral datasets for comprehensive generalization validation, and developing a real-time prediction system for clinical applications.

In the future, integrating pediatric clinical data, such as immune function and treatment history, will provide a more comprehensive view of the factors influencing changes in viral load. Additionally, optimizing the model for long-term forecasting and extending its applicability to real-time clinical settings will be key to its widespread deployment in pediatric infectious disease management. By expanding the verification scope across multiple heterogeneous datasets and adapting the model to various pediatric viral infections, the DTR-Former can serve as a valuable tool for intelligent monitoring and early warning systems for pediatric viral diseases, supporting clinical decision-making, disease prevention, and public health security.

## Data Availability

The original contributions presented in the study are included in the article/supplementary material, further inquiries can be directed to the corresponding author.
